# Improving the Sustainability of Enzymatic Synthesis of Poly(butylene adipate)-Based Copolyesters: Polycondensation Reaction in Bulk vs Diphenyl Ether

**DOI:** 10.1021/acsomega.4c00814

**Published:** 2024-09-04

**Authors:** Martyna Sokołowska, Kristof Molnar, Judit E. Puskas, Miroslawa El Fray

**Affiliations:** † Szczecin, Faculty of Chemical Technology and Engineering, Department of Polymer and Biomaterials Science, West Pomeranian University of Technology, Al. Piastow 45, 70-311 Szczecin, Poland; ‡ Department of Food, Agricultural and Biological Engineering, College of Food, Agricultural and Environmental Science, The Ohio State University, 1680 Madison Avenue, Wooster, Ohio 44691, United States; § Laboratory of Nanochemistry, Department of Biophysics and Radiation Biology, Semmelweis University, Nagyvarad ter 4, Budapest 1089, Hungary

## Abstract

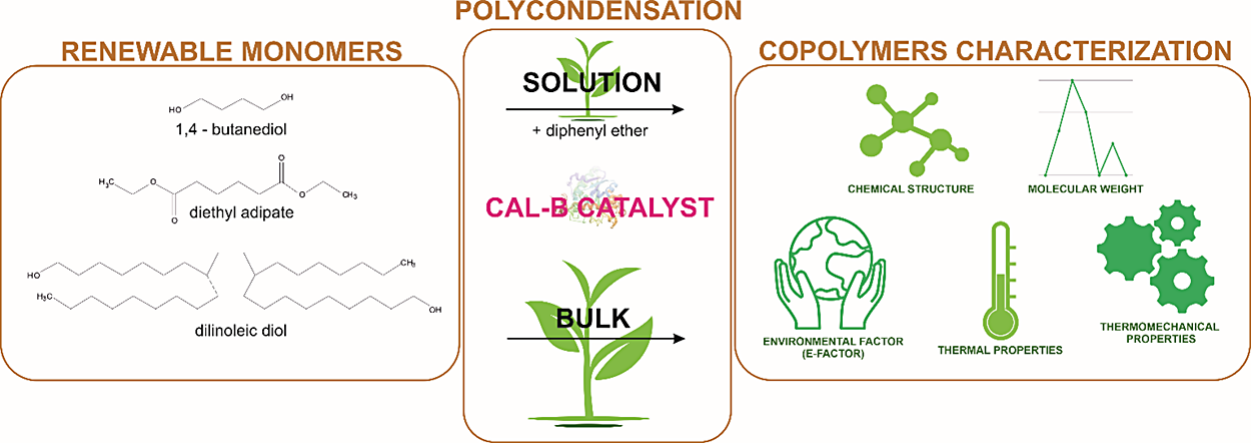

In response to mounting global concerns such as CO_2_ emissions, environmental pollution, and the depletion of fossil resources, the field of polymer science is shifting its focus toward sustainability. This research investigates the synthesis of poly(butylene adipate)-co-(dilinoleic adipate) (PBA-DLA) copolymers using two distinct methods: bulk polycondensation and polycondensation in diphenyl ether. The objective is to assess the environmental impact, chemical structure, composition, and key properties of the resulting copolymers, with a particular emphasis on determining the viability of bulk synthesis as a more sustainable approach. Various analytical methods, including nuclear magnetic resonance spectroscopy, Fourier transform infrared spectroscopy, and size exclusion chromatography, were employed to confirm successful copolymerization and highlight differences in molecular weight and microstructure. Additionally, thermal and dynamic mechanical analyses were conducted to thoroughly characterize the copolymers’ properties. This research provides significant findings into the sustainable production of PBA-DLA copolymers, offering a more environmentally friendly approach without compromising product quality or performance.

## 1 Introduction

In light of the current global situation, which is connected to increasing CO_2_ emissions, environmental pollution, and increasing concerns regarding the depletion of fossil resources, there has been a significant shift toward sustainable practices in the field of polymer science. Consequently, there is now an intensified emphasis on incorporating green chemistry principles into polymer research. By reducing the utilization of hazardous chemicals, minimizing waste generation, and promoting the use of renewable resources, we can actively contribute to a more sustainable future and this approach no longer requires previous justifications to validate its relevance.

One promising avenue of investigation has been the synthesis of biobased polyesters, which has garnered considerable attention in recent years.^1−3^ Polyesters are materials that offer versatility, chemical resistance, and ease of processing, making them suitable for a wide range of applications. They exhibit good dimensional stability, mechanical properties, and UV stability, while their recyclability and aesthetic options make them highly desirable in industries such as textiles, packaging, and automotive.^4,5^ Polyesters have been developed using a variety of compounds derived from biomass, such as building blocks or monomers.^6,7^ Adipic acid and its derivatives as well as aliphatic linear diols such as 1,4-butanediol which were specifically used in this work are one of the top renewable building blocks which have been widely used in polyester synthesis.^8−12^ However, plant oils, as well as fatty acid derivatives, have proved to be a useful basis for developing biobased monomers.^13^ In our previous study, we demonstrated that dilinoleic diol (DLD) possessing a long aliphatic chain (C36) is a promising starting compound of novel polymeric materials.^14−20^ Besides being an almost readily available, natural fatty acid derivative obtained via linoleic and oleic acid dimerization,^21^ it can be used as a comonomer to produce polymeric materials with improved flexibility.^22^

In order to promote a sustainable and environmentally friendly future for the chemical industry, we need to shift our focus beyond just monomers and also prioritize the choice of catalysts and synthesis efficiency. There are two commonly used methods for synthesizing polyesters: ring-opening polymerization of cyclic esters and polycondensation reactions. The traditional polycondensation method involves the polymerization of aliphatic monomers using metal-based catalysts at elevated temperatures.^23^ However, this method can suffer from major drawbacks, such as product discoloration, degradation caused by high temperatures, lack of selectivity, and difficulty in removing residual metals from the final product.^24^ As a result, there has been a growing interest in developing more eco-friendly alternatives to metal-based catalysts, such as organocatalysts and enzymes, which have their own advantages and limitations.

Focusing on enzymatic catalysts as a potential substitute for traditional metal-based catalysts, *Candida antarctica* lipase B (CAL-B) immobilized on the acrylic resin is the most popular due to its eco-friendliness, recyclability, and high selectivity.^25−28^ The last of the mentioned features results from the unique structure of the active site pocket of CAL-B which contributes to its enantio-, regio-, and stereoselectivity and in consequence leads to the formation of products with well-defined structures.^18^ There is a vast body of literature describing the use of enzymes for catalyzing reactions, particularly in the synthesis of polyesters. Among the different types of polyesters, numerous studies have focused on the production of 2,5-furandicarboxylic acid-based,^29,30^ vegetal oil-based,^31^ and sugar-based^32^ polyesters. A large attention was also given to succinate-based^14,33,34^ and adipate-based polyesters.^35−38^

Based on the above-mentioned information, it is evident that enzymes are widely acknowledged for their catalytic efficiency and selectivity in chemical synthesis. However, their industrial application is often hindered by challenges related to scalability and sustainability. One significant challenge in enzyme-based reactions, particularly in polycondensation reactions for polyester synthesis using enzymes like CAL-B, arises from the presence and selection of solvent. Despite the common use of buffered water as a solvent in many enzymatic reactions, CAL-B’s sensitivity to aqueous environments poses limitations, impacting its efficacy in polycondensation. Consequently, achieving optimal enzyme activity and reaction efficiency often demands strictly anhydrous conditions, necessitating the use of organic solvents. This challenge not only carries environmental implications but also contributes to energy consumption and costs due to the purification and precipitation steps involved in obtaining the final product. Even if the solvent is recovered after synthesis, additional energy is required. Therefore, there is a necessity to explore new, more sustainable approaches that can simultaneously result in a lower Environmental factor (E-factor). E-factor is a simple, yet reliable measure used to estimate the efficiency of production by comparing the amount of waste generated to the quantity of product created.^39^

One potential solution to minimize solvent usage and reduce energy consumption is performing solvent-free enzymatic synthesis in bulk. However, it is important to note that this approach also has certain limitations. The mild reaction temperatures required for enzymatic catalysis can lead to high viscosity and reduced fluidity within the reaction medium, making it difficult for reactants to move and hindering the progression of the reaction. Therefore, this method may only be successful when the synthesized polyesters have relatively low melting points, ensuring that the products remain molten during the reaction while preserving enzyme activity. Additionally, according to literature data, enzymatic synthesis in solvents such as diphenyl ether often yields materials with higher molecular weights, making this procedure preferable in many cases36^34,35^ While the bulk polycondensation method, compared to polycondensation in solvents, exhibits a lower E-factor, indicating reduced material consumption, energy requirements, and waste generation, it is crucial to ensure that the final product meets the required specifications. Therefore, the synthesis routes need to be evaluated not only from an environmental standpoint but also in terms of the quality and performance of the resulting material.

In this paper, we will investigate whether synthesis in bulk can provide a poly(butylene adipate)-*co*-(dilinoleic adipate) (PBA-DLA) copolymer with similar or improved properties compared to polycondensation in a solvent. By analyzing the physical and chemical properties of the copolymers produced by both methods, we can determine if bulk synthesis is not only more environmentally friendly but also a viable alternative that can meet the desired product specifications. This research can provide valuable information into the development of sustainable and efficient synthetic routes for the production of copolymers.

## 2 Experimental Section

### 2.1 Materials

The following chemicals were purchased from Sigma-Aldrich: diphenyl ether (DE; ≥99%), Dulbecco’s Phosphate Buffered Saline (DPBS), lipase from *Pseudomonas cepacia* (≥30 U/mg), sodium azide (≥99.5%). Diethyl adipate (DA; ≥99%) was ordered from Matrix Chemicals (Sevelen, Switzerland). 1,4-Butanediol (BDO; ≥99%) was ordered from Alfa Aesar (Kandel, Germany). Dimer linoleic diol (DLD; ≥96.5%) (trade name: Pripol 2033) was provided by Cargill Bioindustrial (Gouda, The Netherlands). Chloroform (≥98.5%) was purchased from Chempur (Piekary Slaskie, Poland) and methanol (≥99.8%) was ordered from Stanlab (Lublin, Poland). Tetrahydrofuran (containing 0.025% butylated hydroxytoluene as a preservative) was purchased from Fisher Chemical, Waltham, MA. polystyrene (PS) standard (Pressure chemicals Lot 80317: *M*w 30,000; *M*_w_/*M*_n_ = 1.06). *Candida antarctica* lipase B (CAL-B) covalently immobilized on polyacrylate beads (300–500 μm; ≥95%, Fermase CAL-B 10,000), with a nominal activity of 10,000 PLU/g (propyl laurate units per gram dry weight) was acquired from Fermenta Biotech Ltd., Mumbai and Enzyme Catalyzed Polymers LLC (Akron, OH, USA). CAL-B was predried under vacuum for 24h at 40 °C and diphenyl ether was stored over 4 Å molecular sieves before use.

### 2.2 CAL-B Catalyzed Polycondensation in Bulk (PBA-DLA_B)

The copolyester of poly(butylene adipate)-*co*-(dilinoleic adipate) (PBA-DLA) with 70–30 wt % hard to soft segment ratio was synthesized *via* two-stage polycondensation method in bulk using CAL-B as biocatalyst. In this experiment, CAL-B (10% of total monomers, 0.82 g), BDO (21.0 mmol, 1.89 g), DA (23.7 mmol, 4.80 g), and DLD (2.77 mmol, 1.50 g) were added to a round-bottom flask and heated in an oil bath with a magnetic stirrer. The reaction was carried out under inert gas flow at atmospheric pressure and an initial temperature of 80 °C. After one hour, the temperature was slowly increased to 95 °C, and the collection of ethanol was monitored for three hours. Then, the reaction was conducted under a pressure of 600 Torr for 21 h, after which the pressure was reduced to 2 Torr while maintaining the temperature at 95 °C for 35 h. At the end of the reaction, the product mixture was dissolved in acetone, filtered to remove CAL-B, and then added dropwise to cold methanol while stirring to precipitate a white polymer product. In the final step, the product was collected, washed with methanol, and dried in a vacuum at 40 °C for 24 h. The copolymer obtained through the described synthesis was designated as PBA-DLA_B.

### 2.3 CAL-B Catalyzed Polycondensation in Diphenyl Ether (PBA-DLA_S)

The copolyester of poly(butylene adipate)-*co*-(dilinoleic adipate) (PBA-DLA) with 70–30 wt% hard to soft segment ratio was synthesized via two-stage polycondensation method in diphenyl ether using CAL-B as biocatalyst according to the protocol described in our previous paper,^40^ however, herein acetone was used as a solvent at the end of the reaction to facilitate the filtration of CAL-B and the precipitation of the final product. The amounts of monomers and catalyst used for the reaction were equal to those employed in the bulk polycondensation method to ensure the most comparable results and to carry out the synthesis under consistent conditions. PBA-DLA synthesized in diphenyl ether is abbreviated as PBA-DLA_S.

### 2.4 Size Exclusion Chromatography (SEC)

SEC measurements were performed using a system consisting of an Agilent 1260 infinity isocratic pump, a Wyatt Eclipse DUALTEC separation system, an Agilent 1260 infinity variable wavelength detector (UV), a Wyatt OPTILAB T-rEX interferometric refractometer, a Wyatt DAWN HELOS-II multiangle static light scattering detector (MALS) with a built-in dynamic light scattering (DLS) module, a Wyatt ViscoStar-II viscometer, an Agilent 1260 infinity standard autosampler, and 6 StyragelVR columns (HR6, HR5, HR4, HR3, HR1, and H0.5). The columns were thermostatted at 35 °C and tetrahydrofuran (THF), continuously distilled from CaH_2_, was used as the mobile phase at a flow rate of 1 mL/min. The results were analyzed using the ASTRA 7 software (Wyatt Technology, Santa Barbara, CA, USA). As quality control, a polystyrene standard (*M*_w_ 30,000; *M*_w_/*M*_n_ = 1.06.) was injected and analyzed using a d*n*/d*c* = 0.185 mL/g. Since the d*n*/d*c* was unknown for the PBA-DLA_B and S samples, 100% mass recovery was assumed. The software calculated the d*n*/d*c* value for each sample. Samples were first dissolved in THF from the distillation to obtain 3–4 mg/mL solutions then 1 mL was filtered into the SEC vials using a 0.45 μm PTFE syringe filter and submitted to the machine. In each case, 100 μL was injected.

### 2.5 Nuclear Magnetic Resonance Spectroscopy (NMR)

^1^H and ^13^C NMR spectra of PBA-DLA were recorded using a Bruker spectrometer (800 MHz, 10 s relaxation delay, 128 scans for ^1^H NMR and 700 MHz, 10 s relaxation delay, 5120 scans for ^13^C NMR). The samples were dissolved in CDCl_3_, and tetramethylsilane (TMS) was used as the internal reference. The NMR results obtained in this study were analyzed using MestreNova 2009 and Origin 2021 software.

### 2.6 Attenuated Total Reflection Fourier Transform Infrared (FTIR) Spectroscopy

Spectra of vacuum-dried samples were acquired using a Bruker ALPHA spectrometer with diamond ATR crystal. The spectra were recorded over a spectral range of 400–4000 cm^–1^ with a resolution of 2 cm^–1^, and 32 scans were performed for each sample.

### 2.7 Thermal Properties (DSC)

The thermal properties of the materials were evaluated using a TA Instruments DSC Q2500 Discovery differential scanning calorimeter (DSC). The samples were heated and cooled at a rate of 10 °C/min, and the measurements were performed over a temperature range of −90 to 200 °C in a nitrogen atmosphere. The glass transition temperature (*T*_g_) was determined as the midpoint of the transition observed during a second heating step.

### 2.8 Thermomechanical Properties (DMTA)

Dynamic mechanical and thermal analysis (DMTA) was conducted on samples produced through melt-pressing at a temperature of 60 °C, resulting in specimens that were 100 μm thick, 10 mm wide, and 50 mm long. Using a DMA Q800 device from TA Instruments, measurements were performed in tensile mode. The analysis was conducted with a constant frequency of 1 Hz, a heating rate of 2 °C/min, and an amplitude of 60. This allowed for accurate and precise determination of the desired properties.

## 3 Results and Discussion

Polycondensation of diethyl adipate, 1,4–butanediol, and dilinoleic diol in the presence of CAL-B was conducted in diphenyl ether and in bulk. The impact of the polymerization method was assessed in terms of E-factor, copolymer chemical structure, composition, yield by weight (%), number, and weight averaged molecular weight (*M*_n_ and *M*_w_, respectively), as well as thermal and thermomechanical properties.

To assess the environmental impact of the enzymatic synthesis process conducted under different conditions, namely in bulk and diphenyl ether, the E-factor values were estimated using eqs 1 and 2. Reagent calculations were performed to obtain 6 g of PBA-DLA copolyesters. The reaction consisted of 1,4-butanediol (21.0 mmol, 1.89 g), diethyl adipate (23.7 mmol, 4.80 g), and dilinoleic diol (2.77 mmol, 1.50 g) in diphenyl ether (96.22 mol, 16.38 g), which yielded 5.28 g of PBA-DLA_S (88% by weight). Alternatively, the reaction conducted in bulk (without diphenyl ether) resulted in 5.72 g of PBA-DLA_B (95% by weight). Additionally, calculations considered the amount of tetrahydrofuran (THF) used for CAL-B purification for subsequent reaction cycles to ensure possibly comprehensive results. Table 1 presents the amount of waste generated during the reaction, CAL-B filtration, and purification, as well as product purification and precipitation.

**Table 1:** Calculation of PBA-DLA_S and PBA_DLA_B Reaction Total Waste

	weight [g]	
waste	PBA-DLA_S	PBA-DLA_B
unreacted monomers/purification-related polymer loss	0.72	0.28
ethanol (byproduct)	2.18	2.18
diphenyl ether	16.38	
acetone	35.28	11.76
methanol	285.12	95.04
THF	39.96	39.96
**total amount of waste**	**379.64**	**149.22**
total amount of waste after THF and methanol recovery*	**54.56**	**14.22**


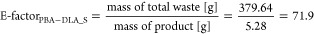
1


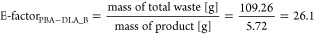
2

Obtained results indicate that bulk polycondensation leads to lower waste generation per unit mass of the product compared to polycondensation carried out in diphenyl ether. The E-factor for the bulk process is approximately three times lower than that of the solvent-based process (26.1 and 71.9 for PBA-DLA_B and PBA-DLA_S, respectively). This suggests that the bulk polycondensation method is more resource-efficient and environmentally friendly, generating significantly less waste per unit mass of the final product. Moreover, additional calculations of the E-factor, including THF and methanol recovery, were performed. It is important to note that such recovery processes involve additional energy consumption. Therefore, while the E-factor calculation presented here offers a preliminary insight into the potential environmental impact of the reaction, a detailed Life Cycle Analysis is crucial for a comprehensive understanding. This analysis should consider various factors such as the manufacture of biobased monomers, enzyme production, and overall energy consumption. Nevertheless, eqs 3 and 4 present E-factor calculations after methanol and THF recovery, resulting in values of 2.5 and 10.3 for PBA-DLA_S and PBA-DLS_S, respectively. Those values, further underscore the environmentally friendly nature of the bulk synthesis method.
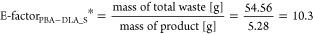
3
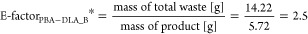
4

**Figure 1:**
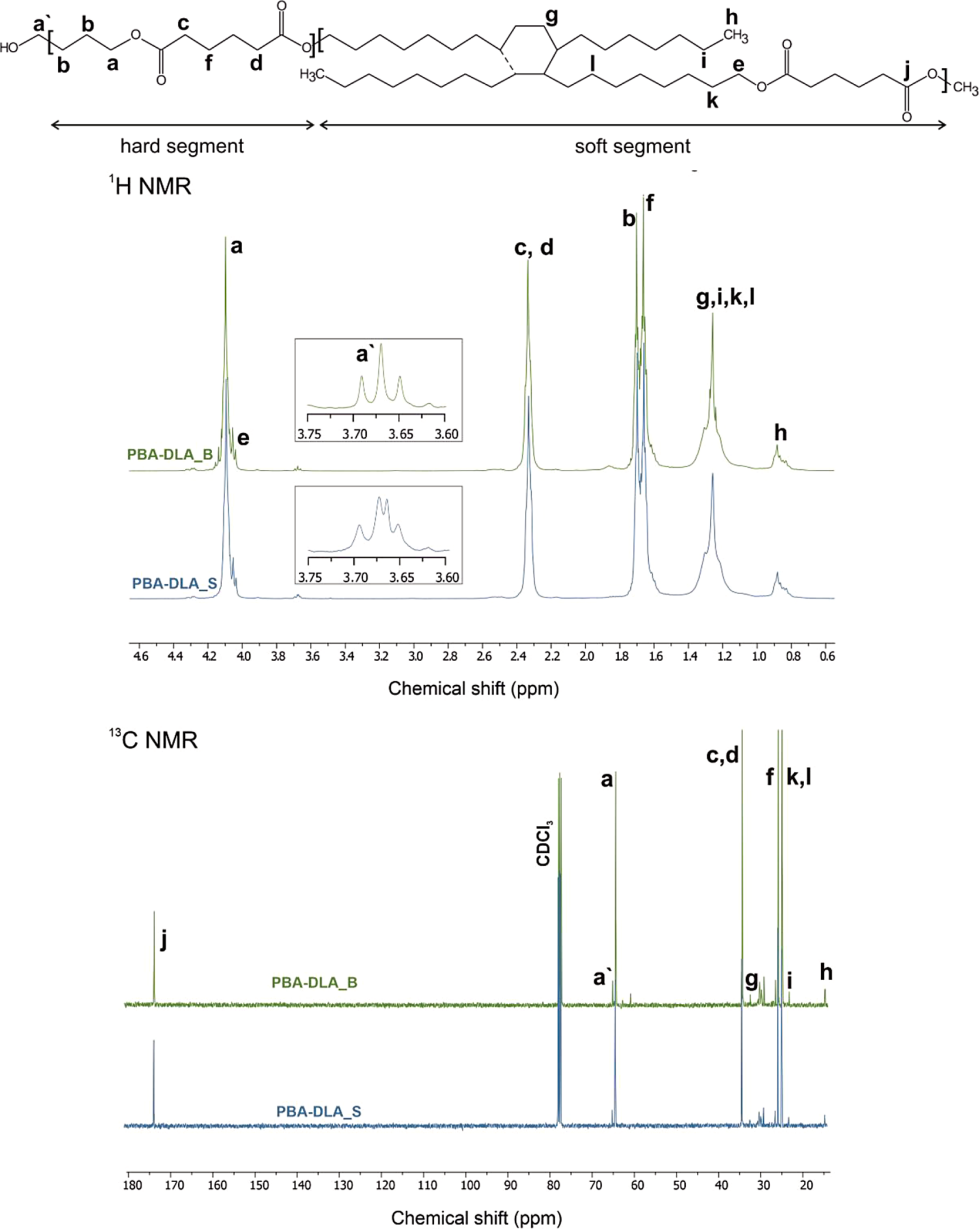
^1^H NMR and ^13^C NMR spectra of the poly(butylene adipate)-co-(dilinoleic adipate) copolyesters.

Furthermore, to assess the chemical structure of copolyesters, NMR and FTIR analyses were carried out. The ^1^H NMR and ^13^C NMR spectra, which include detailed NMR assignments are presented in Figure 1. NMR assignments are ascribed as follows: ^1^H NMR (400 MHz, CDCl_3_, ppm): 4.09 (4H,–CO–O–CH_2_, from BDO), 4.05 (4H,–CO–O–CH_2_, from DLD), 3.68 (4H,–CH_2_–OH, end group from BDO and DLD), 2.33 (4H,–CO–O–CH_2_, from DA), 1.70 (4H,–CO–O–CH_2_–CH_2_, from BDO), 1.66 (4H,–CO–O–CH_2_–CH_2_, from DA), 1.30–1.26 (−CH_2_–internal methylene groups from DLD), 0.88 (6H,–CH_2_–CH_3_, end groups from DLD). ^13^C NMR (400 MHz, CDCl_3_, ppm): 173.27 (−C=O–O–, from DA), 64.80 (−CH_2_–OH, end group from BDO and DLD), 64.08 (−CO–O–CH_2_, from BDO), 33.61 (−CO–O–CH_2_, from DA), 32.12, 25.12, 24.36 (internal −CH_2_– from DLD), 14.10 (−CH_2_–CH_3_, end groups from DLD).

The ^1^H and ^13^C NMR spectra of PBA-DLA copolyesters showed characteristic resonances corresponding to the structures illustrated in Figure 1, specifically, resonances attributed to the newly formed adipic-butanediol (A-BDO) and adipic-dilinoleic (A-DLD) diads, which proved the success of PBA-DLA copolytransesterification reaction.

Based on the ^1^H NMR signals characteristic for hard and soft segments, real segmental composition with number averaged molecular weight (*M*_n_) were calculated according to the method described in Supporting Information and presented in Table 2.

**Table 2:** Composition of PBA-DLA Copolyesters Determined from ^1^H NMR and SEC^b^

	composition: wt % [mol %]		^1^H NMR^c^			SEC^a^^,^^d^		
copolymer	theoretical	calculated^c^	*M*_n_ [g/mol]	*M*_n_ [g/mol]	*M*_w_ [g/mol]	*Đ*	oligomer content [wt %]	DP
**PBA-DLA_S**	70/30 [88.4/11.6]	67/33 [87.1/12.9]	19 900	14 400	70 300	4.88	6.2	55.7
**PBA-DLA_B**		68/32 [87.2/12.8]	29 800	23 100	50 800	2.20	6.0	89.8

a *M*_n_ – number average molecular mass, *M*_w_ – weight average molecular mass, *Đ* – dispersity index.

b DP–degree of polymerization calculated as a ratio of the number averaged molecular weight of copolymer and molecular weight of PBA and DLA repeating units (see SI for detailed calculations).

c Values calculated from^1^H NMR.

d Values determined by SEC.

As evidenced by Table 2, the final compositions of PBA-DLA copolymers are comparable to the initial values. In both cases, there is a greater content of soft segments, which may be due to the removal of BDO when the high vacuum is applied. Since the reaction is performed with stoichiometric quantities, the absence of BDO leads to lower hard segment content. The rest of the reagents have higher boiling points, including DLD which due to its long aliphatic chain, is difficult to evaporate under high vacuum conditions.

**Figure 2:**
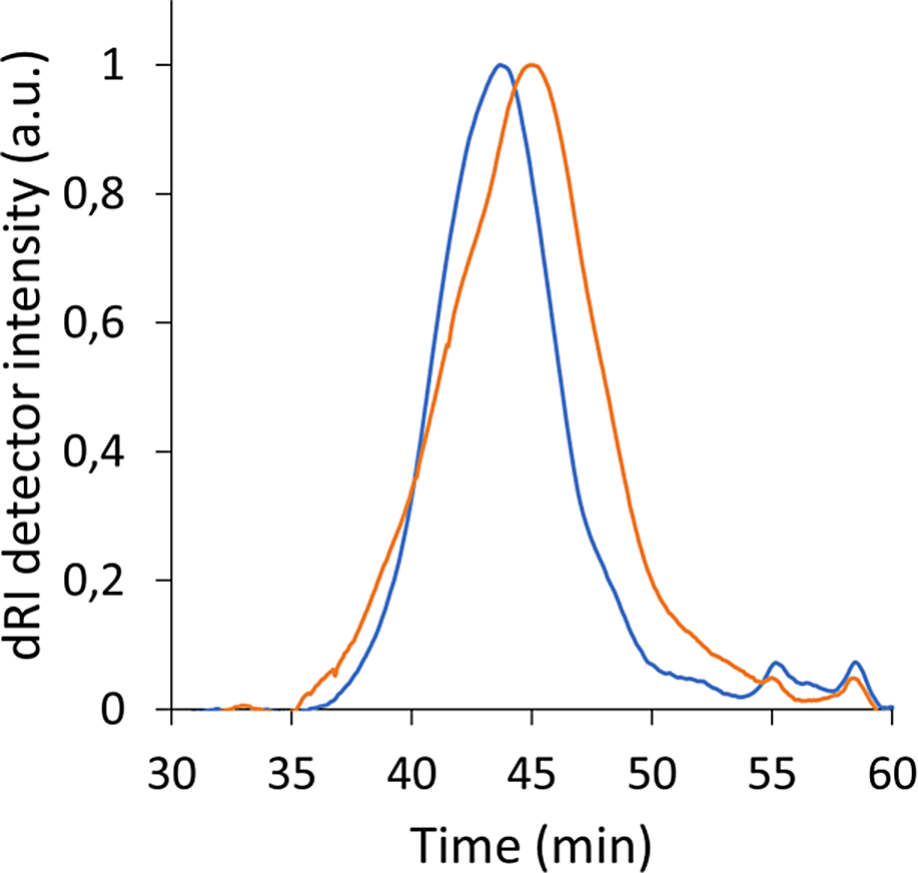
SEC dRI signal vs elution time: PBA-DLA_S (orange) and PBA-DLA_B (blue). Chromatograms are normalized to enable better visual comparison.

The SEC chromatogram of the two polymers are very similar (see Figure 2): there is a main polymer peak and two small peaks at longer elution times marked with the black arrows. The peak at 55 min is DLA and the other at ∼58 min is probably another oligomer from the synthesis (see Supporting Information Figure S5). PBS-DLS_S possesses lower *M*_n_ values compared to PBA-DLA_B (14,400 vs 23,100 g/mol, respectively), which is in accordance with the *M*_n_ values calculated from ^1^H NMR. Nevertheless, PBA-DLA_S has a higher *M*_w_ (70,300 g/mol) compared to that of PBA-DLA_B (50,800 g/mol), whereas its dispersity (*Đ*) is much higher as well, with a value of 4.88 compared to 2.20 (see Table 2). These alterations in *Đ* values may be attributed to the differences in the copolymer’s microstructure and block arrangements, which result from the synthesis conditions and CAL-B selectivity. (see Table 3).

**Table 3:** Degree of Randomness and Sequence Segment Length Calculated from ^13^C NMR

copolymer	*R*^a^	*L*_BDO-DA_^b^	*L*_DLD-DA_^b^
**PBA-DLA_S**	0.80	4.79	1.68
**PBA-DLA_B**	1.02	2.82	1.49

a Degree of randomness calculated from eq 4.

b Average sequence length calculated from eqs 2 and 3.

**Figure 3:**
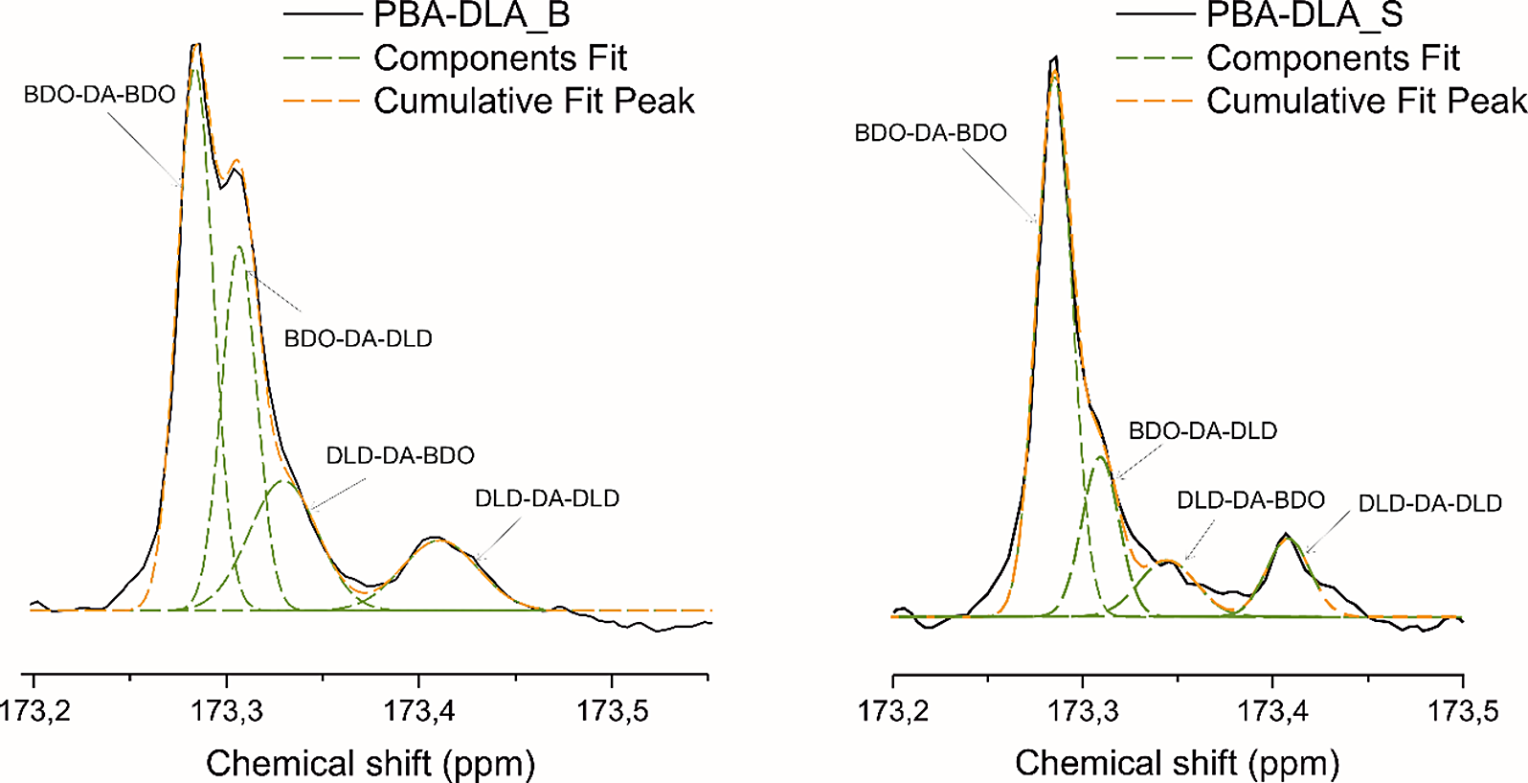
^13^C NMR spectrum of the PBA-DLA_B and PBA-DLA_S carbonyl carbon region after deconvolution with the possible arrangements and combinations of the four triads corresponding to each peak.

Moreover, an analysis of the segmental distribution within the copolyester microstructure was carried out to gain a deeper understanding of the microstructure of the copolymers. This analysis revealed changes in the chemical environment of the signal observed at δ^13^C = 173.3 ppm, which corresponds to the carbonyl carbon atoms, due to the presence of BDO or DLD. These changes result in four potential monomer sequence variations (BDO-DA-BDO, BDO-DA-DLD, DLD-DA-BDO, DLD-DA-DLD), providing meaningful data into the copolymer’s structural characteristics (Figure 3).

Upon deconvolution of the signals corresponding to the characteristic carbon atoms of carbonyl groups between two hard segments (BDO–DA–BDO; 173.29 ppm), two soft segments (DLD–DA–DLD; 173.41 ppm), and hard–soft segments (DLD–A–BDO/BDO–DA–DLD; 173.31–173.35 ppm), we evaluated the molecular architecture. This assessment included determining the degree of randomness (*R*) and the average sequence length of the hard and soft segments (*L*_BDO–DA_, *L*_DLD–DA,_ respectively). These parameters were calculated using eqs 5–7.^41^
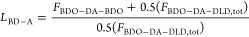
5
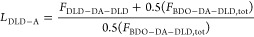
6
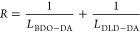
7where *F_x_* is the normalized integral value from ^13^C NMR (*x* = BDO–DA–DLD,BDO-side; BDO–DA–DLD,DLD-side; BDO–DA–BDO; DLD–DA–DLD). The results calculated for each copolymer are presented in Table 3.

When the degree of randomness (*R*) is 1, it indicates that the copolymer segments are randomly distributed. However, when the *R* values are lower than 1, it suggests that the segments tend to cluster in blocks.

Based on the data presented in Table 3, it is evident that while the *L*_DLD–DA_ values are comparable for PBA-DLA_S and PBA-DLA_B, there is a notable difference in the *L*_BDO–DA_ values. Both copolymers have a longer BDO–DA sequence, however, in PBA-DLA_S, it is twice as long, indicating a more distinct blocky distribution within the macromolecule. This observation is further supported by the calculated *R* values, with the *R* value of PBA-DLA_S being lower than 1, indicating a blocky segmental distribution, while the *R* value of PBA-DLA_B is closer to unity, suggesting a relatively more random chemical structure. A potential explanation for this phenomenon is the high selectivity of CAL-B toward the monomers used in the synthesis. When the synthesis is performed in solution, CAL-B’s increased mobility allows it to more readily interact with the monomer toward which it exhibits higher catalytic activity. In this case, CAL-B may exhibit enhanced activity toward BDO-DA due to its shorter aliphatic chain, which can reach the active site pocket of CAL-B more easily than DLD possessing a long aliphatic chain (C = 36). As a result, during solution-phase reactions, the formation of BDO-DA blocks may take precedence, serving as the primary product. Subsequently, when BDO monomer is consumed, DLD-DA sequences are formed in a second order, and in the end, the copolyester microstructure is more blocky. On the other hand, when the synthesis is carried out in bulk, it restricts enzyme mobility due to increased viscosity, causing BDO-DA and DLD-DA sequences to be catalyzed randomly. Similar results were provided by Ilarduya and co-workers,^42^ where enzymatic polycondensation of copolyester materials in bulk led to products with a random microstructure.

**Figure 4:**
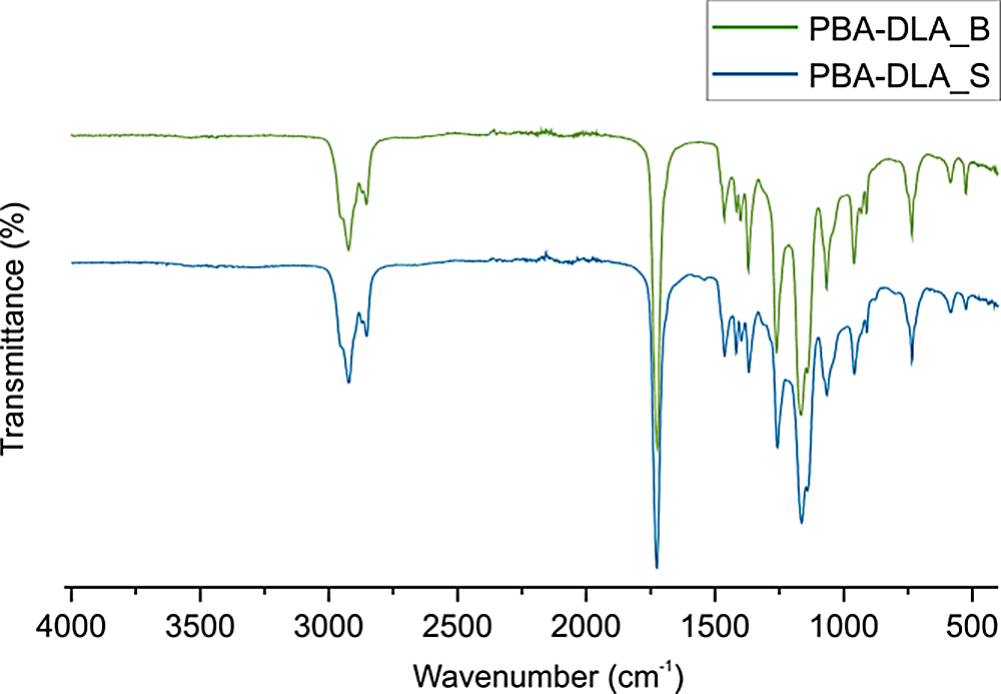
ATR-FTIR spectra of PBA-DLA 70–30 copolyesters.

Chemical structure of resulting copolyesters was also assessed using Fourier transform infrared (FTIR) spectrocopy (Figure 4).

Analysis of the FTIR spectra revealed the presence of characteristic functional groups in PBA-DLA 70–30 copolyesters. Two distinct peaks at 2923 and 2853 cm^–1^ were observed and were attributed to the asymmetric and symmetric stretching vibrations of the −CH_2_– groups in the soft DLA sequences, respectively. The carbonyl C=O stretching vibrations were represented by a strong band at 1726 cm^–1^, while the ester C–O–C groups were identified by the peaks at 1257 and 1163 cm^–1^, corresponding to the asymmetric and symmetric stretching vibrations, respectively. The deformation and wagging vibrations of the methylene −CH_2_– groups were identified by multibands appearing at 1417–1463 cm^–1^ and 1369–1398 cm^–1^, respectively. Additionally, the in-plane and out-of-plane deformation vibrations of aliphatic C–H and C–C groups were represented by bands in the 1000–500 cm^–1^ region. It is worth mentioning that no significant differences were observed in the region 1700–500 cm^–1^ for the copolymer series, which is where the most important functional groups are found.

**Figure 5:**
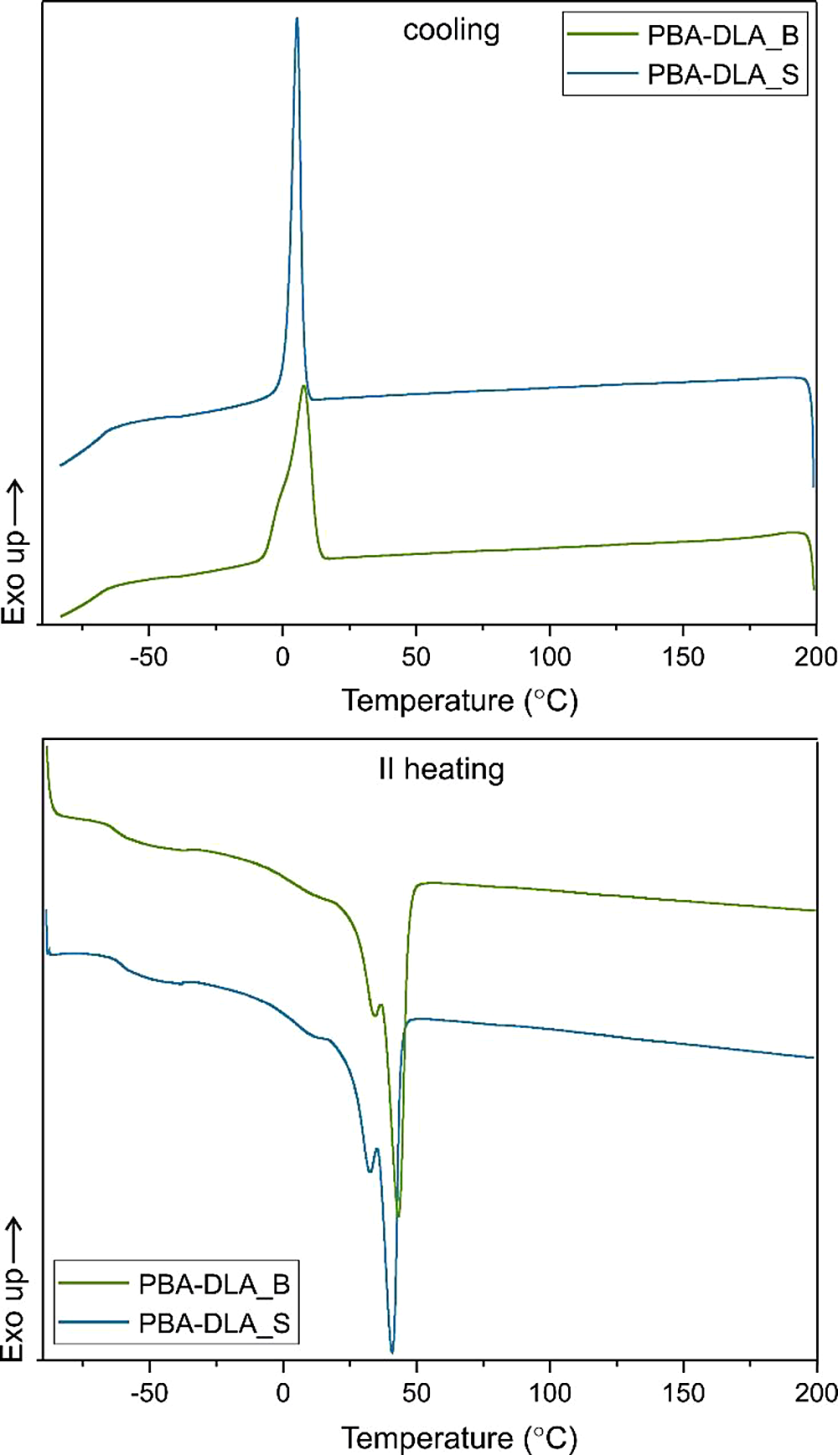
DSC cooling and second heating thermograms of PBA-DLA copolymers.

To further evaluate the phase transition temperatures and their thermal effects, copolymer samples were subjected to DSC analysis. A heating–cooling–heating cycle was performed as described in the Materials and Methods section, with only the cooling and second heating measurements being considered for comparison. The total crystalline phase content (*X*_c,tot_) and crystalline phase content in the hard segments (*X*_c,h_) were calculated using eqs 8 and 9, respectively.
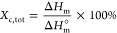
8
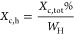
9where *W*_H_ represents the weight content of the hard segments (PBA), Δ*H*_m_ is the melting enthalpy of the copolymer (PBA-DLA), and Δ*H*_m_° is the melting enthalpy of 100% crystalline PBA (135.0 J/g).^43^ The DSC thermograms and numerical values obtained are presented in Figure 5 and Table 4, respectively.

**Table 4:** DSC Results for PBA-DLA Copolymer Series^a^

copolymer	*T*_g_ [°C]	Δ*C*_p_ [J/g*·*°C]	*T*_δ_ [°C]	*T*_c_ [°C]	Δ*H*_c_ [J/g]	*T*_m_ [°C]	Δ*H*_m_ [J/g]	*X*_c,h_ [%]	*X*_c,tot_ [%]
**PBA-DLA_S**	–61	0.270	–42	6	43.8	40	47.6	52.7	35.3
**PBA-DLA_B**	–62	0.244	–47	9	44.9	42	46.0	50.1	34.1

a *T*_g_ – glass transition temperature; *T*_δ_ – glass transition temperature from DMTA (determined as max. of tan δ); Δ*C*_p_ – heat capacity at *T*_g_; Δ*H*_m_ – melting enthalpy of the hard segments; *T*_m_ – melting temperature; *T*_c_ – crystallization temperature; *X*_c,h_ – crystalline phase content in the hard segment phase; *X*_c,tot_ – total crystalline phase content in the polymer. Both *X*_c,h_ and *X*_c,tot_ were computed using the actual Wh segment as determined via ^1^H NMR.

Based on DSC analysis, it was evident that PBA-DLA copolyesters exhibited a semicrystalline nature, characterized by distinct *T*_g_, *T*_m_, and *T*_c_ transitions. Focusing on the *T*_g_ values, they provide clear evidence of the successful integration of amorphous DLA segments within the rigid PBA matrix. Both copolyesters displayed similar *T*_g_ values, with slightly lower temperatures observed for the PBA-DLA_B copolyester (−61 and −62 °C for PBA-DLA_S and PBA-DLA_B, respectively). Furthermore, upon cooling the molten copolyester, the resulting crystalline morphologies exhibited a crystallization transition at 6 and 9 °C for PBA-DLA_S and PBA-DLA_B, respectively. In the case of PBA-DLA_S, crystallization begins at lower temperature rates due to higher *M*_w_ values. Polymers with higher molecular weights tend to crystallize more slowly since larger molecules have more complex structures and are more difficult to arrange in an ordered pattern. Furthermore, referring to the crystallization degree, PBA-DLS_S copolymer possesses slightly higher *X*_c,h,_ and *X*_c,tot_ values than PBA-DLA_B which may be attributed to the differences in copolymer microstructure. PBA-DLA_S possesses a longer average sequence length of hard segments (*L*_BDO-DA_) which may result in more efficient formation of the crystalline phase due to stronger intra- and/or intermolecular interactions between rigid sequences. Obtained copolyesters are characterized by relatively low melting temperatures (40 and 42 °C for PBA-DLA_S and PBA-DLA_B, respectively), and therefore, the range of possible applications is rather limited. However, they can be successfully used in other fields, for example in biomedical applications as drug nanocarriers as we demonstrated in our previous work.^16^

**Figure 6:**
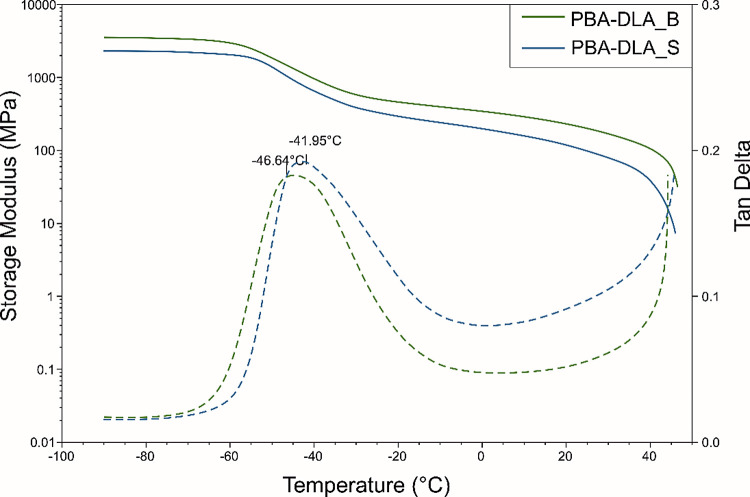
DMTA traces of the PBA-DLA copolyesters

The effect of the synthesis route on the dynamic mechanical properties of PBA-DLA copolyesters was monitored in the tensile mode at a starting temperature of −90 °C. Isochronal evolution of the storage modulus with temperature as well as temperature dependence of tan δ for PBA-DLA copolyesters is presented in Figure 6.

Recorded dynamic mechanical thermal analysis (DMTA) data provided information on the viscoelastic properties of copolyesters. It is evident that the storage modulus (*E′*) remains constant at temperatures below the glass transition temperature. Subsequently, a noticeable decrease in *E′* values is observed when the δ transition (*T*_δ_) occurs within the amorphous phase. For both PBA-DLA_B and PBA-DLA_S, *T*_δ_ falls within the range of −47 to −42 °C, respectively. Notably, PBA-DLA_B exhibits lower *T*_δ_ values, which can be correlated with material microstructure, as well as its lower crystallinity degree values (*X*_c,tot_, *X*_c,h_) determined from DSC (Table 4), which translates to an increased elastic response. Furthermore, in the case of PBA-DLA_B, the average sequence length of hard segments (*L*_BDO-DA_) is approximately half as short as that of PBA-DLA_S, hence, molecular motions in PBA-DLA_B are probably less restricted by hard crystalline domains. The obtained results also reveal that despite having lower values of *M*_w_, PBA-DLA_B exhibits a higher *E′* value. This phenomenon can be attributed to the lower dispersity index (see Table 2), signifying a narrower molecular weight distribution which results in more consistent packing and entanglement of polymer chains, ultimately enhancing the material’s load-bearing capabilities.

## 4 Conclusions

In this research article, we conducted a comprehensive investigation into the synthesis of poly(butylene adipate)-co-(dilinoleic adipate) (PBA-DLA) copolymers using two distinct methods: bulk polycondensation and polycondensation in diphenyl ether. Our study aimed to evaluate the environmental impact, chemical structure, composition, and key properties of the resulting copolymers, ultimately determining the viability of bulk synthesis as a more sustainable and environmentally friendly approach.

Our results demonstrate that bulk polycondensation emerges as the promising method in terms of environmental sustainability. The E-factor analysis revealed a striking reduction in waste generation per unit mass of the final product for bulk synthesis compared to the solvent-based method. This signifies a substantial decrease in resource consumption, energy requirements, and waste generation when employing the bulk approach.

NMR and FTIR spectroscopy confirmed the successful copolymerization of PBA-DLA in both synthesis methods, yielding copolymers with the expected chemical structures and characteristic functional groups. Additionally, through SEC and ^13^C NMR deconvolution, we observed differences in molecular weight and microstructure between the two synthesis routes. Bulk synthesis produced copolymers with a more random microstructure, while the solution-phase synthesis yielded more blocky copolymers.

Furthermore, thermal properties assessed using DSC analysis revealed that both synthesis methods yielded semicrystalline copolymers with similar transition temperatures and crystallinity degree. Moreover, DMTA provided insight into the viscoelastic properties of the copolymers. Notably, we observed that bulk-synthesized copolymers exhibited better load-bearing capabilities despite their lower molecular weight, probably owing to narrower molecular weight distribution and more consistent chain packing.

The findings of this study underscore the significant advantages of bulk polycondensation as a sustainable and efficient method for producing PBA-DLA copolymers. This environmentally friendly approach not only reduces waste but also yields copolymers with properties comparable to material synthesized in a solvent. Future research may explore tailored synthesis conditions to further enhance the properties of copolymers synthesized via bulk methods, opening up new avenues for sustainable materials with enhanced performance characteristics.

In conclusion, our research provides valuable information about sustainable polymer synthesis, highlighting bulk enzymatic polycondensation as a promising pathway toward a more eco-friendly and efficient future for copolymer production. These findings hold relevance for industries seeking to adopt greener practices while maintaining product quality and performance.

## Supporting Information Available

Raw data from NMR, SEC, and DSC analysis (PDF)

## Abbreviations

^13^C NMR: carbon nuclear magnetic resonance
^1^H NMR: proton nuclear magnetic resonance
*Đ*: dispersity index
BDO: 1,4-butanediol
CAL-B: *Candida antarctica* lipase type B
DE: diphenyl ether
DFA: dimerized fatty acid
DMTA: dynamic thermos-mechanical analysis
DSC: differential scanning calorimetry
FTIR: Fourier Transform Infrared Spectroscopy
*M*_n_: number averaged molecular weight
*M*_w_: weight averaged molecular weight
PBA-DLA: poly(butylene adipate)-*co*-(dilinoleic adipate)
SEC: size exclusion chromatography
*T*_c_: crystallization temperature
*T*_g,_: glass transition temperature
*T*_m_: melting temperature
Δ*H*_m_: melting enthalpy
Δ*H*_m_°: melting enthalpy of fully crystalline polyester
Δ*C*_p_: heat capacity
