# Supplementary material for: Improving the Sustainability of Enzymatic Synthesis of Poly(butylene adipate)-Based Copolyesters: Polycondensation Reaction in Bulk vs Diphenyl Ether
Source: ACS Omega. 2024 Sep 4;9(37):38385–95. doi: 10.1021/acsomega.4c00814 (PMC11411551; doi:10.1021/acsomega.4c00814)
Supplement: Supplementary file 1 — ao4c00814_si_001.pdf [file ao4c00814_si_001.pdf]

Supporting information

Improving the sustainability of enzymatic synthesis of poly(butylene adipate)-based copolyesters. Polycondensation reaction in bulk vs in diphenyl ether.

Martyna Sokołowska[1], Kristof Molnar[2,3], Judit E. Puskas[2], Miroslawa El Fray[1]*

[1]West Pomeranian University of Technology, Szczecin, Faculty of Chemical Technology and Engineering, Department of Polymer and Biomaterials Science, Al. Piastow 45, 71-311 Szczecin, Poland

[2]Department of Food, Agricultural and Biological Engineering, College of Food, Agricultural and Environmental Science, The Ohio State University, 1680 Madison Avenue, Wooster, OH 44691ristof, Judit

[3]Laboratory of Nanochemistry, Department of Biophysics and Radiation Biology, Semmelweis University, Nagyvarad ter 4. Budapest, Hungary 1089;

*Corresponding author: mirfray@zut.edu.pl

CONTENTS

^{1}H NMR and ^{13}C NMR 2

Molecular mass calculation from ^{1}H NMR 4

SEC 7

Degree of polymerization calculation from SEC 8

DSC 10

^{1}H NMR and ^{13}C NMR

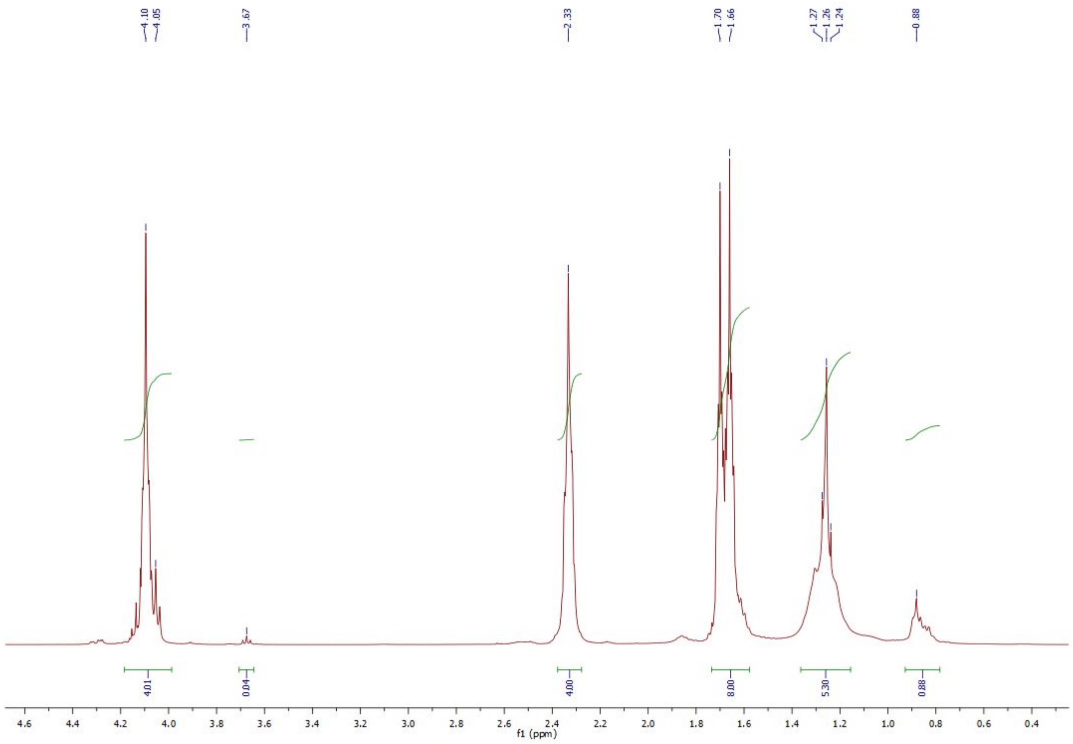

Figure S1. ^{1}H NMR spectra of PBA-DLA_B

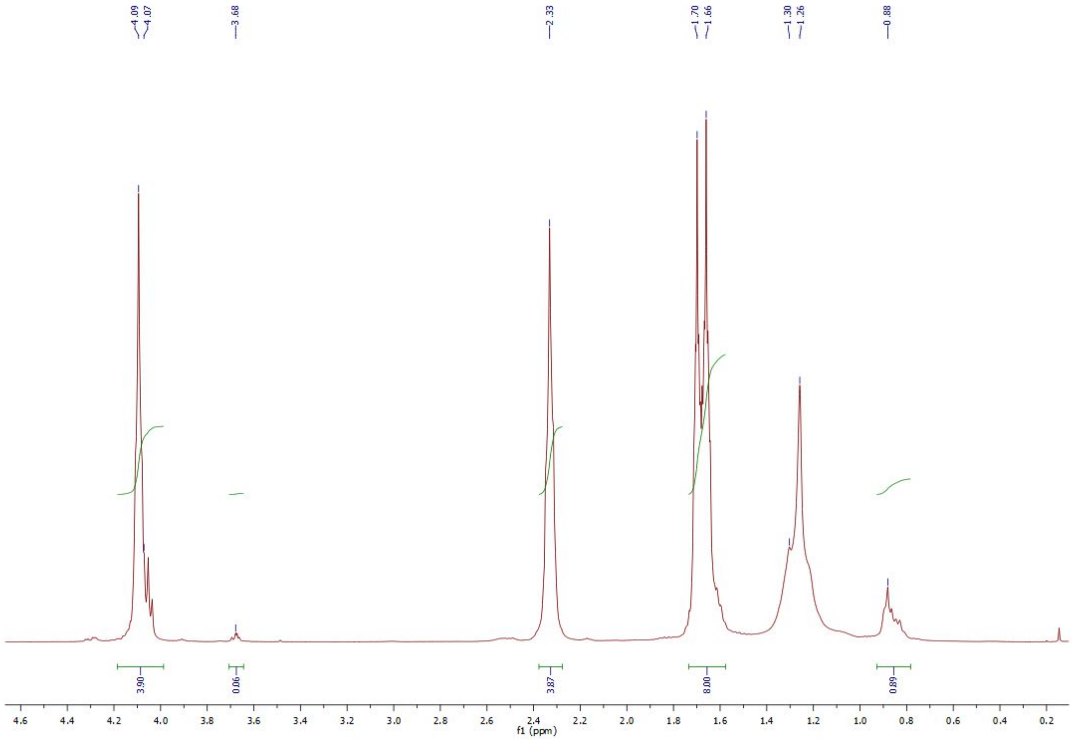

Figure S2. ^{1}H NMR spectra of PBA-DLA_S.

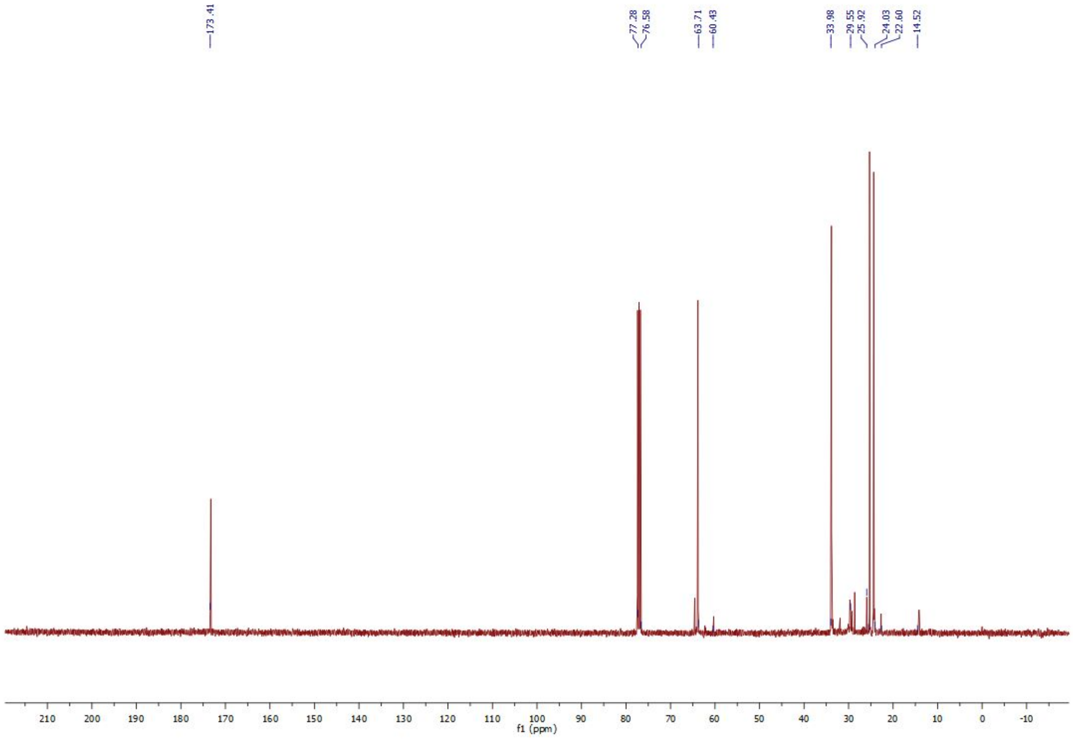

Figure S3. ^{13}C NMR spectra of PBA-DLA_B

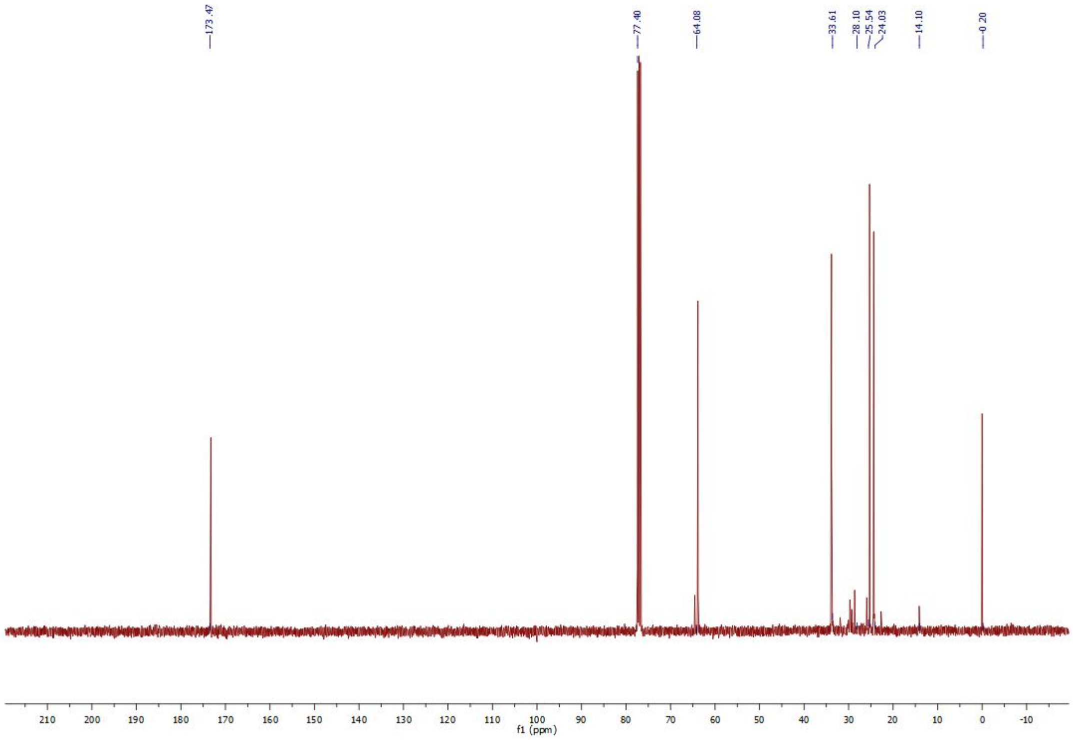

Figure S4. ^{13}C NMR spectra of PBA-DLA_S

PBA-DLA_B – molar calculations

Reagent	M.W.	ppm	Hydrogens	Integration	H/species	Wt.	Wt. %	Mol %	M_n [g/mol]
PBA	200.00	1.70	4.00	4.00	1.00	200.0	67.65	87.21	29 400
DLA	652.00	0.88	6.00	0.88	0.15	95.6	32.35	12.79	
End-group		3.68	2.00	0.04					

PBA-DLA_S – molar calculations

Reagent	M.W.	ppm	Hydrogens	Integration	H/species	Wt.	Wt. %	Mol %	M_n [g/mol]
PBA	200.00	1.70	4.00	4.00	1.00	200.0	67.41	87.08	19 900
DLA	652.00	0.88	6.00	0.89	0.15	96.7	32.59	12.92	
End-group		3.68	2.00	0.06					

Molecular mass calculation from ^{1}H NMR

Since the peak at 1.70 ppm (b) is related to four protons in the BDO sequence (-CH_2-) of PBA segment and the peak at 0.88 ppm (g) is arising from six protons in DLA unit (-CH_3-), H was computed following the equation:

$$H = \frac{\frac{I_{1.70}}{n_{1.70}}}{\frac{I_{0.88}}{n_{0.88}}} \qquad (1)$$

H is the ratio between hard and soft segments, $I_{1.70}$ is the integral of the signal at 1.70 ppm and $I_{0.85}$ is the integral of signal at 0.85 ppm. The weight percentage of hard segments ($\%W_h$) of PBA-DLA copolyester was computed from H using equation (2):

$$\%W_h = \frac{H \cdot M_h}{H \cdot M_h + M_s} \cdot 100\% \qquad (2)$$

where $\%W_H$ is the weight percentage of PBA hard segments, M_h is the molecular weight of the hard segment (200 g/mol) and M_s is the molecular weight soft segment (652 g/mol). Likewise, using the signals of BDO and DLD, we can also compute the mole percent of each block using equations (3-4):

$$[\%Mol_h] = \frac{\frac{I_{1.70}}{n_{1.70}}}{\frac{I_{1.70}}{n_{1.70}} + \frac{I_{0.88}}{n_{0.88}}} \cdot 100\% \qquad (3)$$

$$[\%Mol_s] = \frac{\frac{I_{0.88}}{n_{0.88}}}{\frac{I_{1.70}}{n_{1.70}} + \frac{I_{0,88}}{n_{0,88}}} \cdot 100\% \qquad (4)$$

where $\%Mol_h$ and $\%Mol_s$ are mol percentage of hard and soft segments, respectively. $n_{1,70}$ and $n_{0,88}$ are number of protons in BDO and DLA units.

Following the equations (5-6) we can also calculate the number of hard and soft segments (Num_h and Num_s) by comparing the BDO and DLD signals to that of the end groups, which are signals arising from hydroxyl end-groups of BDO at 3.68 ppm ($-CH_2O$**H**) and the macromolecules can be expected to be capped by a BDO on either end, so there will be two such end-groups (n_{end}).

$$[Num_h] = \frac{(I_{1.70} \cdot n_{3.68} \cdot n_{end})}{I_{3.68} \cdot n_{1.70}} \qquad (5)$$

$$[Num_s] = \frac{(I_{0.88} \cdot n_{3.68} \cdot n_{end})}{I_{3.68} \cdot n_{0.88}} \qquad (6)$$

Finally, by multiplying the number of each block times the molecular weight of each block and summing, we are able to calculate molecular weight of copolymer using equation (7):

$$[M_n] = Num_h \cdot M_h + Num_s \cdot M_s \qquad (7)$$

Calculations for PBA-DLA_B

$$H = \frac{\frac{I_{1.70}}{n_{1.70}}}{\frac{I_{0.88}}{n_{0.88}}} = \frac{\frac{4}{4}}{\frac{0.89}{6}} = 6.82$$

$$\%W_h = \frac{H \cdot M_h}{H \cdot M_h + M_s} \cdot 100\% = \frac{6.82 \cdot 200}{6.82 \cdot 200 + 652} \cdot 100\% = 67.66\%$$

$$[\%Mol_h] = \frac{\frac{I_{1.70}}{n_{1.70}}}{\frac{I_{1.70}}{n_{1.70}} + \frac{I_{0.88}}{n_{0.88}}} \cdot 100\% = \frac{\frac{4}{4}}{\frac{4}{4} + \frac{0.88}{6}} \cdot 100\% = 87.21$$

$$[\%Mol_s] = \frac{\frac{I_{0.88}}{n_{0.88}}}{\frac{I_{1.70}}{n_{1.70}} + \frac{I_{0,88}}{n_{0,88}}} \cdot 100\% = \frac{\frac{0.88}{6}}{\frac{4}{4} + \frac{0.88}{6}} \cdot 100\% = 12.79$$

$$[Num_h] = \frac{(I_{1.70} \cdot n_{3.68} \cdot n_{end})}{I_{3.68} \cdot n_{1.70}} = \frac{(4 \cdot 2 \cdot 2)}{0.04 \cdot 4} = 100$$

$$[Num_s] = \frac{(I_{0.88} \cdot n_{3.68} \cdot n_{end})}{I_{3.68} \cdot n_{0.88}} = \frac{(0.88 \cdot 2 \cdot 2)}{0.04 \cdot 6} = 15$$

$$[M_n] = Num_h \cdot M_h + Num_s \cdot M_s = 100 \cdot 200 + 15 \cdot 652 = 29\,400 \text{ g/mol}$$

Calculations for PBA-DLA_S

$$H = \frac{\frac{I_{1.70}}{n_{1.70}}}{\frac{I_{0.88}}{n_{0.88}}} = \frac{\frac{4}{4}}{\frac{0.88}{6}} = 6.74$$

$$\%W_h = \frac{H \cdot M_h}{H \cdot M_h + M_s} \cdot 100\% = \frac{6.74 \cdot 200}{6.74 \cdot 200 + 652} \cdot 100\% = 67.41\%$$

$$[\%Mol_h] = \frac{\frac{I_{1.70}}{n_{1.70}}}{\frac{I_{1.70}}{n_{1.70}} + \frac{I_{0.88}}{n_{0.88}}} \cdot 100\% = \frac{\frac{4}{4}}{\frac{4}{4} + \frac{0.89}{6}} \cdot 100\% = 87.08$$

$$[\%Mol_s] = \frac{\frac{I_{0.88}}{n_{0.88}}}{\frac{I_{1.70}}{n_{1.70}} + \frac{I_{0,88}}{n_{0,88}}} \cdot 100\% = \frac{\frac{0.89}{6}}{\frac{4}{4} + \frac{0.89}{6}} \cdot 100\% = 12.92$$

$$[Num_h] = \frac{(I_{1.70} \cdot\ n_{3.68} \cdot n_{end})}{I_{3.68} \cdot\ n_{1.70}} = \frac{(4 \cdot 2 \cdot 2)}{0.06 \cdot 4} = 67$$

$$[Num_s] = \frac{(I_{0.88} \cdot\ n_{3.68} \cdot n_{end})}{I_{3.68} \cdot\ n_{0.88}} = \frac{(0.89 \cdot 2 \cdot 2)}{0.06 \cdot 6} = 10$$

$$[M_n] = Num_h \cdot M_h + Num_s \cdot M_s = 67 \cdot 200 + 10 \cdot 652 = \text{ 19 900 g/mol}$$

SEC

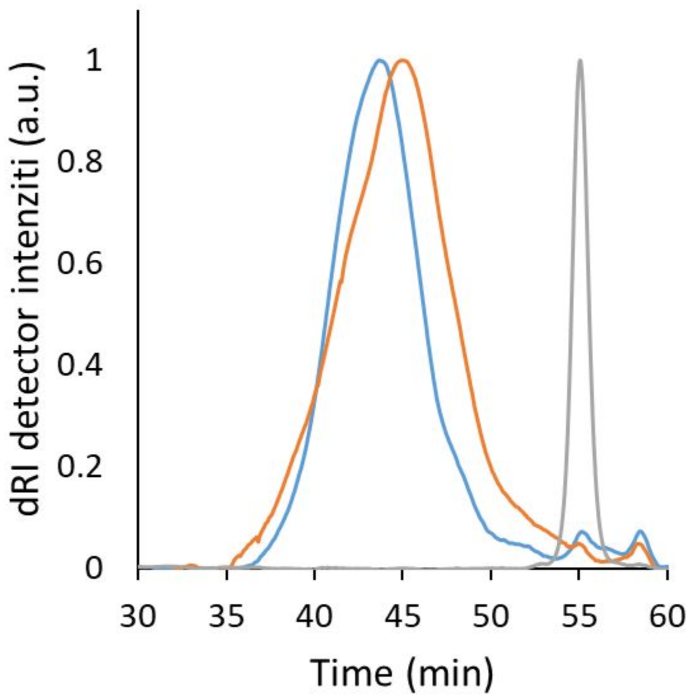

Figure S5. SEC dRI signal vs elution time: PBA-DLA_S (orange), PBA-DLA_B (blue) and DLA (grey). Chromatograms are normalized for enabling better visual comparison.

Degree of polymerization calculation from SEC

In order to calculate the degree of polymerization (DP) for copolymers, which contain two different repeating units (hard and soft segments), consideration of the composition of the copolymer was required. The degree of polymerization was calculated for each repeating unit

separately, and then a weighted average was calculated based on the composition of the copolymer assessed via ^{1}H NMR analysis.

General approach to calculate the DP for a copolymer with two different repeating units:

For calculations, weight fractions of each monomer unit were denoted as f_1 and f_2, where $f_1 + f_2 = 1$. Degree of polymerization for hard and soft segments were further calculated using equations (8), and (9), respectively.

$$DP_H = \frac{M_n}{M_h} \qquad (8)$$

$$DP_S = \frac{M_n}{M_S} \qquad (9)$$

Where DP_H and DP_S is the degree of polymerization of hard and soft segment, respectively. M_n is the number averaged molecular weight of copolymer acquired from SEC measurement (14 400 g/mol), and M_H and M_S is the molecular weight of PBA hard (200 g/mol), and DLA soft segment (652 g/mol), respectively.

Furthermore, averaged degree of polymerization (DP) was calculated based on equation (10):

$$DP = f_1 \cdot DP_H + f_2 \cdot DP_S \qquad (10)$$

This calculation provides an average degree of polymerization for the copolymer, taking into account the different repeating units and their respective proportions in the polymer chain.

Calculations for PBA-DLA_S

$$DP_H = \frac{M_n}{M_h} = \frac{14\,400}{200} = 72$$

$$DP_S = \frac{M_n}{M_S} = \frac{14\,400}{652} = 22$$

$$DP = f_1 \cdot DP_H + f_2 \cdot DP_S = 0.6741 \cdot 72 + 0.3259 \cdot 22 = 55.7$$

Calculations for PBA-DLA_B

$$DP_H = \frac{M_n}{M_h} = \frac{23\,100}{200} = 116$$

$$DP_S = \frac{M_n}{M_S} = \frac{23\,100}{652} = 35$$

$$DP = f_1 \cdot DP_H + f_2 \cdot DP_S = 0.6765 \cdot 116 + 0.3235 \cdot 35 = 55.71 = 89.8$$

DSC

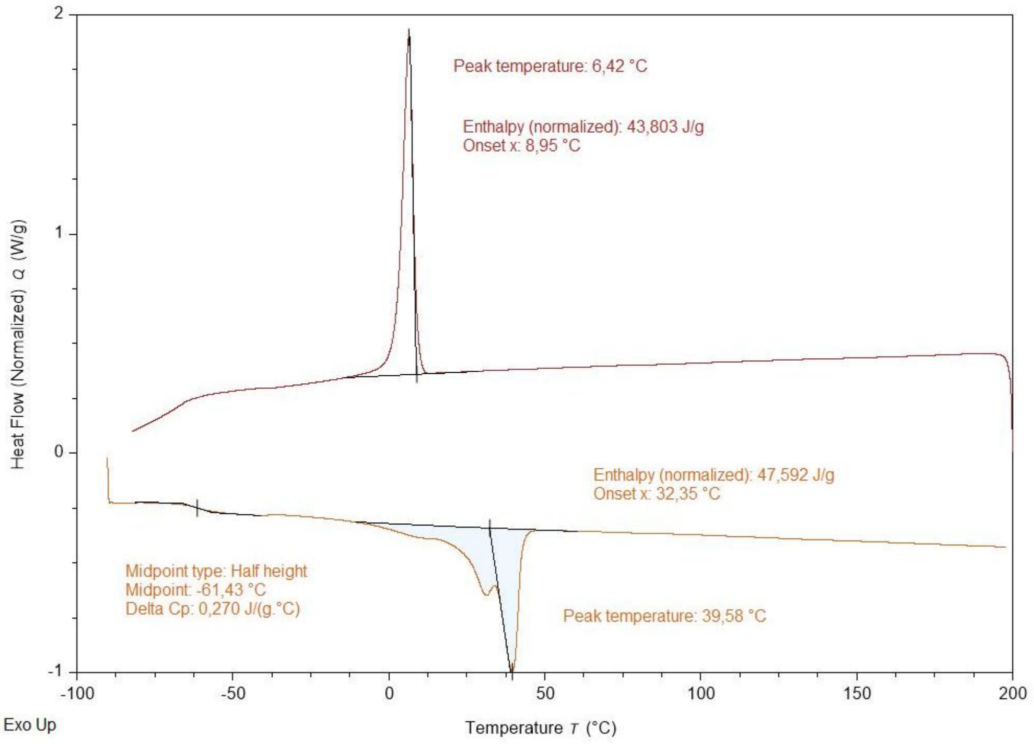

Figure S6. DSC thermograms for PBA-DLA_S copolyester.

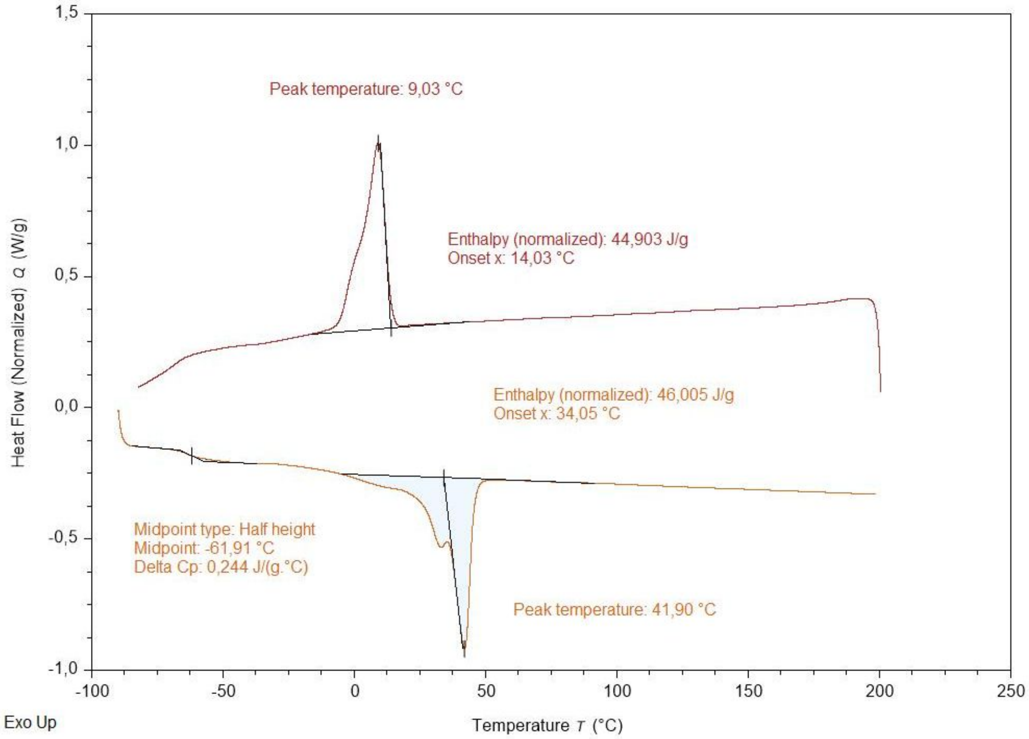

Figure S7. DSC thermograms for PBA-DLA_B copolyester
